# S/N Co-Doped Ultrathin TiO_2_ Nanoplates as an Anode Material for Advanced Sodium-Ion Hybrid Capacitors

**DOI:** 10.3390/molecules29184507

**Published:** 2024-09-23

**Authors:** Yuzhu Li, Qing Lan, Yuanfei Gao, Dan Zhang, Guangyin Liu, Jinbing Cheng

**Affiliations:** 1 College of Chemistry and Pharmaceutical Engineering, Nanyang Normal University, Nanyang 473061, China; lanqnynu@163.com (Q.L.); gaoyf8810@163.com (Y.G.); dazhangny@163.com (D.Z.); 2 Henan International Joint Laboratory of MXene Materials Microstructure, College of Physics and Electronic Engineering, Nanyang Normal University, Nanyang 473061, China; chengjinbing1988@163.com

**Keywords:** sodium-ion capacitors, TiO_2_ nanoplates, sulfur and nitrogen co-doping, high electrochemical kinetics

## Abstract

Anatase titanium dioxide (TiO_2_) has emerged as a potential anode material for sodium-ion hybrid capacitors (SICs) in terms of its nontoxicity, high structure stability and cost-effectiveness. However, its inherent poor electrical conductivity and limited reversible capacity greatly hinder its practical application. Here, ultrathin TiO_2_ nanoplates were synthesized utilizing a hydrothermal technique. The electrochemical kinetics and reversible capacity were significantly improved through sulfur and nitrogen co-doping combined with carbon coating (SN-TiO_2_/C). Sulfur and nitrogen co-doping generated oxygen vacancies and introduced additional active sites within TiO_2_, facilitating accelerated Na-ion diffusion and enhancing its reversible capacity. Furthermore, carbon coating provided stable support for electron transfer in SN-TiO_2_/C during repeated cycling. This synergistic strategy of sulfur and nitrogen co-doping with carbon coating for TiO_2_ led to a remarkable capacity of 335.3 mAh g^−1^ at 0.1 A g^−1^, exceptional rate property of 148.3 mAh g^−1^ at 15 A g^−1^ and a robust cycling capacity. Thus, the SN-TiO_2_/C//AC SIC delivered an impressive energy density of 177.9 W h kg^−1^. This work proposes an idea for the enhancement of reaction kinetics for energy storage materials through a synergistic strategy.

## 1. Introduction

Lithium-ion batteries (LIBs) have become the predominant energy storage solution in portable electronics and electric vehicles. However, the escalating demand for renewable energy storage applications is restricted by the growing scarcity of lithium resources, which is making it necessary to explore alternatives. Sodium-based energy storage devices demonstrate significant potential for renewable energy storage grids, primarily attributed to the uniform distribution and low cost of sodium resources [1,2]. Sodium-ion capacitors (SICs), which synergistically combine the high energy density characteristic of Na-ion batteries with the rapid power output of supercapacitors, have garnered considerable attention as potential candidates for advanced energy storage systems [3,4]. The performance of SICs heavily depends on the performance of the anode material. Nevertheless, the increased ionic radius and inherently slower diffusion efficiency of Na^+^ compared to Li^+^ lead to substantial challenges, resulting in undesirable electrochemical performance [5]. Various anodes have been explored aimed at improving the Na-ion storage capability of SICs, including carbonaceous materials, [6,7,8] transition metal sulfides/phosphides [9,10,11,12] and alloys [13]. Despite some progress, the development of high-efficiency SICs has so far been limited by the drawbacks of these anode materials. Consequently, an urgent demand exists for anodes with the characteristics of high reversible capacity and extended cycling stability, as well as superior electrochemical kinetics, to advance the performance of SICs.

Among various anode materials, anatase titanium dioxide (TiO_2_) has attracted great interest in terms of its high structure stability, cost-effectiveness and nontoxicity [14]. Additionally, TiO_2_ has a favorable insertion potential of approximately 0.7 V vs. Na/Na^+^, coupled with intercalation pseudocapacitive behavior, which makes it a promising candidate for SICs [15]. Although advantageous, the utilization of TiO_2_ for Na-ion storage is severely restricted by its intrinsic poor electrical conductivity [16].

To address these challenges, researchers have pursued various strategies to boost the Na-ion storage capability of TiO_2_. The fabrication of nanostructured TiO_2_ has demonstrated significant effectiveness in improving Na storage capabilities. Various nanostructures, including nanowires [17], hollow spheres [18], nanoparticles [19], nanotubes [20,21,22], nanoflowers [23], microrods [24,25] and micro-sheets [26] have been studied. Additionally, Shi et al. demonstrated that nanosheet-structured TiO_2_ significantly enhances electrochemical performance due to its increased surface area, which provides abundant Na-ion adsorption sites and shorter Na-ion diffusion distances [27]. However, its reversible capacity and Na-ion reaction kinetics remain undesirable.

Heteroatom doping has emerged as a promising strategy to overcome these limitations by introducing crystal defects and oxygen vacancies, thereby enhancing conductivity and providing abundant active sites for Na-ion storage. For example, sulfur [28,29], nitrogen [16,30], phosphorus [31], silicon [32] and cobalt [33] doping, as well as co-doping [34], have been proven to significantly boost the Na-ion storage capability of TiO_2_. Co-doping, for instance, has been shown to reconstruct charge transfer channels as well as reduce the Na-ion transmission energy barrier [33], while S doping has been reported to promote Na-ion diffusion dynamics in TiO_2_, resulting in an enhanced Na storage capacity and rate performance [28]. Furthermore, nitrogen doping has been substantiated as an effective strategy to enhance the electrical conductivity of TiO_2_, which, in turn, significantly improves its sodium-ion storage capability [35,36]. Therefore, it is vital to modify TiO_2_ with heteroatom doping to attain an extraordinary Na-ion storage performance and enhanced cycle capability. Carbon coating not only establishes efficient electron transfer pathways but also significantly enhances the electrical conductivity of electrodes. For instance, Tang et al. reported that the encapsulation of (NiCo)_3_Se_4_ within a three-dimensional cross-linked carbon network can substantially improve its sodium-ion storage capabilities [37].

Herein, we present a synergistic strategy integrating sulfur and nitrogen co-doping with carbon coating to augment the Na storage capability of TiO_2_ nanoplates. The ultrathin TiO_2_ nanoplates were synthesized utilizing a hydrothermal technique. The synergistic effects of S/N co-doping and carbon coating on the Na-ion storage capabilities of TiO_2_ nanoplates (SN-TiO_2_/C) were systematically investigated. S/N co-doping introduces defects and provides additional active sites, thereby promoting faster Na-ion diffusion and enhancing the overall electrochemical kinetics. Simultaneously, the incorporation of carbon markedly boosts the electrical conductivity of the SN-TiO_2_/C composite, ensuring continuous electron pathways and preventing the agglomeration of SN-TiO_2_/C nanoplates during the sodiation and desodiation cycles. This study presents a comprehensive modification strategy for titanium dioxide, integrating structural control, heteroatom doping, and carbon coating. These approaches work synergistically through multiple mechanisms to markedly enhance the specific capacity and rate performance of SN-TiO_2_/C nanoplates. Owing to the significantly enhanced reaction dynamics and capacity of the SN-TiO_2_/C anode, the fabricated SIC device achieves a high energy density of 177.9 W h kg^−1^. This work integrates structural design, defect engineering and carbon coating strategies to enhance the performance of titanium dioxide, providing valuable insights for the modification of energy storage electrodes.

## 2. Results and Discussion

**Figure 1:**
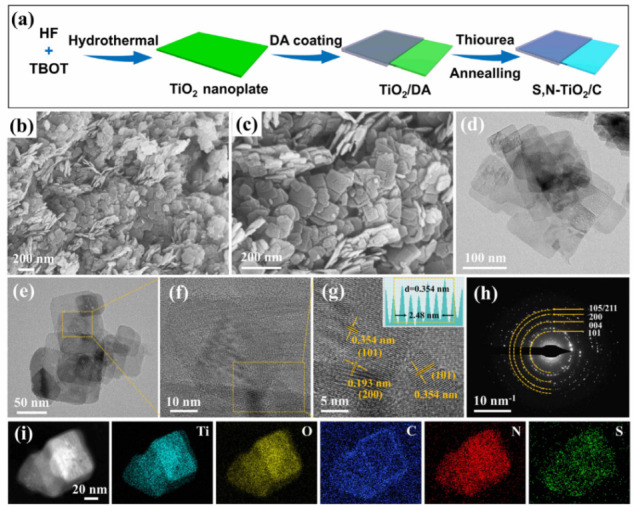
(**a**) Diagrammatic image of the fabrication of SN-TiO_2_/C composite. (**b**,**c**) SEM images of SN-TiO_2_/C. (**d**,**e**) TEM image. (**f**,**g**) HRTEM image and lattice fringes. (**h**) SAED pattern of SN-TiO_2_/C. (**i**) HAADF-STEM image of SN-TiO_2_/C and the elements of Ti, O, C, N, and S.

Figure 1a schematically exhibits the synthesis procedure of SN-TiO_2_/C nanoplates. First, the TiO_2_ nanoplates were produced using a simple hydrothermal technique via the use of titanium butoxide and hydrofluoric acid. Following this, TiO_2_@polydopamine (TiO_2_@PDA) was produced through the self-polymerization of dopamine (DA). The final SN-TiO_2_/C sample was then obtained by annealing TiO_2_@PDA in a thiourea vapor atmosphere. The scanning electron microscope (SEM) images in Figure 1b,c illustrate the morphology of the SN-TiO_2_/C sample. The images reveal a well-defined nanoplate structure. The thin nanoplate structure of SN-TiO_2_/C, which is crucial to offering plentiful Na-ion adsorption sites and reducing diffusion paths for Na-ions, was further confirmed through transmission electron microscope (TEM) analysis (Figure 1d,e). As can be seen, at the edge of the SN-TiO_2_/C nanoplates (Figure S1), there is a graphitized structure in the carbon coating layer. The HRTEM image in Figure 1f,g and SAED pattern in Figure 1h exhibit the distinct lattice fringes of SN-TiO_2_/C, suggesting a high crystallinity. In addition, the lattice spacing of 0.354 nm and 0.193 nm corresponds to the (101) and (200) planes of anatase TiO_2_, respectively. Figure 1i displays the HAADF-STEM images of SN-TiO_2_/C, accompanied by EDS elemental mappings, revealing a uniform dispersion of Ti, O, C, N and S elements.

**Figure 2:**
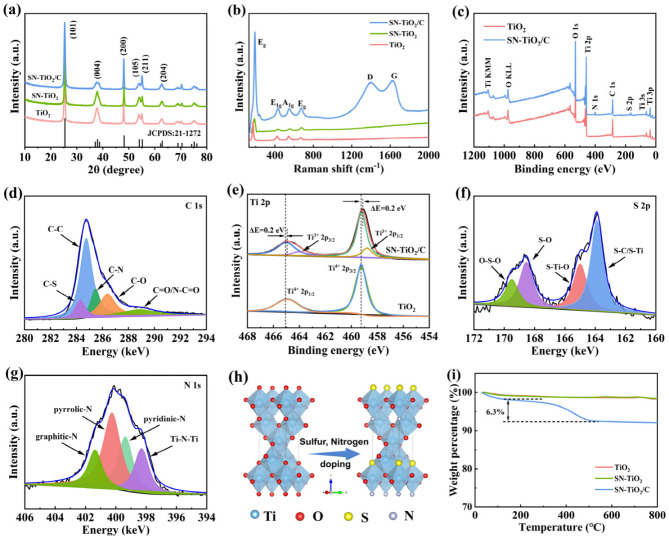
(**a**) XRD patterns of TiO_2_, SN-TiO_2_ and SN-TiO_2_/C. (**b**) Raman profiles of TiO_2_, SN-TiO_2_ and SN-TiO_2_/C. (**c**) XPS survey spectra of TiO_2_ and SN-TiO_2_/C. High-resolution XPS spectra of (**d**) C1s, (**e**) Ti 2p, (**f**) S 2p and (**g**) N 1s of SN-TiO_2_/C. (**h**) Schematic diagram of the structural evolution of sulfur and nitrogen doping into TiO_2_. (**i**) TG curves of TiO_2_, SN-TiO_2_ and SN-TiO_2_/C.

The crystal structures of the TiO_2_, SN-TiO_2_ and SN-TiO_2_/C samples were analyzed using X-ray diffraction (XRD). As depicted in Figure 2a, the sharp diffraction peaks observed for all three samples correspond to the anatase phase of TiO_2_ (JCPDS: 21–1272) and exhibit high crystallinity and purity, indicating that sulfur and nitrogen doping does not change the anatase structure. The distinct peaks at 25.24, 37.76, 48.00, 53.94, 55.00 and 62.54° are ascribed to the (101), (004), (200), (105), (211) and (204) planes of anatase TiO_2_, respectively. Figure 2b depicts the Raman spectra of SN-TiO_2_/C to elucidate their components. The characteristic peaks at 151.6, 391.8, 505.8 and 627.1 cm^−1^ are attributed to the Eg, B1g, A1g and Eg phonon modes of anatase TiO_2_, respectively [20]. Additionally, the D-bond (~1360 cm^−1^) and G-bond (~1577 cm^−1^) observed in the Raman spectra are indicative of disordered carbon and graphitic carbon, respectively [38], confirming the existence of carbon in the SN-TiO_2_/C sample. The D-band to G-band intensity ratio (I_D_/I_G_) in SN-TiO_2_/C is 0.91, suggesting a highly disordered carbon structure.

X-ray photoelectron spectroscopy (XPS) was performed to study the surface chemical states of TiO_2_ and SN-TiO_2_/C. The elements C, N, O, S and Ti were detected in the full survey spectrum of SN-TiO_2_/C (Figure 2c). The S and N contents of SN-TiO_2_/C are 0.65 at% and 1.2 at%, respectively. High-resolution XPS spectra of the C component in SN-TiO_2_/C are shown in Figure 2d. The peaks at 284.3 and 285.5 eV are indicative of the C-S and C-N bonds, revealing the successful incorporation of sulfur and nitrogen atoms into the carbon skeleton [39]. Figure 2e shows the Ti 2p peaks of two samples. In the Ti 2p spectrum for TiO_2_, the observed peaks at 465.1 and 459.3 eV are indicative of the Ti 2p_1/2_ and Ti 2p_3/2_ levels of Ti^4+^, respectively. In the SN-TiO_2_/C sample, the Ti 2p spectrum is deconvoluted into four distinct peaks. The peaks at 464.7 and 459.0 eV are indicative of the Ti 2p_1/2_ and Ti 2p_3/2_ levels of Ti^4+^, which shifted to lower binding energies by 0.2 eV related to the oxygen vacancies in TiO_2_ induced by S and N doping [29,34]. The peaks at 464.1 and 458.8 eV in SN-TiO_2_/C are indicative of the Ti 2p_1/2_ and Ti 2p_3/2_ levels of Ti^3+^, which arise from the reduction in Ti^4+^ to balance the charge due to oxygen vacancies [26]. The observed decrease in binding energy, concomitant with the emergence of Ti^3+^, unequivocally substantiates the emergence of oxygen vacancies. The XPS spectrum of the S component in SN-TiO_2_/C (Figure 2f) shows peaks at 164.9 and 163.8 eV, corresponding to the S-Ti bonds, implying that S is successfully doped into TiO_2_ [29]. The high-resolution XPS spectra of the N 1s component in SN-TiO_2_/C are shown in Figure 2g. The deconvoluted peaks at 401.4, 400.3, and 399.4 eV are indicative of graphitic-N, pyrrolic-N and pyridinic-N, respectively, and the peak at 398.3 eV is associated with Ti-N-Ti bonds, indicating that N-doped TiO_2_ is obtained through replacing lattice oxygen [16]. These findings verify the effective doping of sulfur and nitrogen into the TiO_2_ and carbon structures. Figure 2h exhibits the structural evolution following sulfur and nitrogen doping. The thermogravimetric analysis (TGA) curves of the TiO_2_, SN-TiO_2_ and SN-TiO_2_/C samples (Figure 2i) show a significant mass reduction observed between 400 and 450 °C, indicative of the oxidation of carbon to CO_2_, and the carbon content is 6.3%.

**Figure 3:**
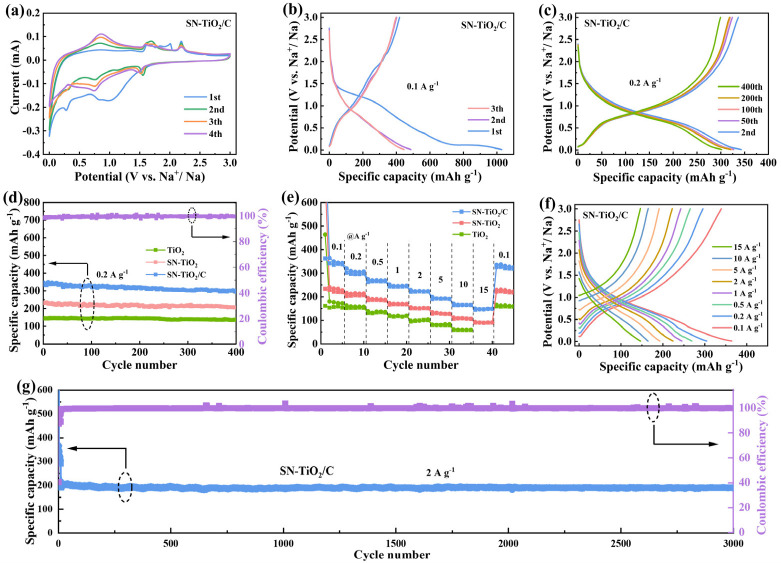
(**a**) CV curves of SN-TiO_2_/C at 0.2 mV s^−1^ (0.01–3.0 V); (**b**) initial GCD profiles of SN-TiO_2_/C at 0.1 A g^−1^; (**c**) GCD curves of SN-TiO_2_/C at 0.2 A g^−1^; (**d**) comparison of cycle ability of TiO_2_, SN-TiO_2_ and SN-TiO_2_/C; (**e**) rate properties of the TiO_2_, SN-TiO_2_ and SN-TiO_2_/C electrodes and (**f**) the corresponding GCD profiles of SN-TiO_2_/C; (**g**) long-term cycle ability of SN-TiO_2_/C electrode.

The Na storage capabilities of the SN-TiO_2_/C electrode were investigated. As revealed in Figure 3a, during the initial cyclic voltammetry (CV) curve, a significantly reduced peak appears around 1.0 V and vanishes in later cycles due to the generation of an SEI film [33]. The prominent cathodic peak that is observed at 0.10 V and appears in all cyclic sweeps is associated with continued electrolyte decomposition [40]. The cathodic peak observed at 0.74 V in the second and subsequent cycles is related to the reduction of Ti^4+^ to Ti^3+^. Moreover, the anode peak at 0.83 V corresponds to the oxidation of Ti^3+^ to Ti^4+^ [26]. At the second and subsequent cycles in the SN-TiO_2_/C electrode, there is a cathodic peak at 1.6 V, along with the two anode peaks at 1.75 V and 2.2 V, which are identified as the stepped redox reactions between S and Na. The CV curves for the SN-TiO_2_/C electrode exhibit sharper and more pronounced redox peaks compared to the TiO_2_ electrode (Figure S2a), suggesting enhanced electrochemical kinetics due to sulfur and nitrogen co-doping and carbon coating. Figure 3b reveals the initial three galvanostatic charge discharge (GCD) profiles of the SN-TiO_2_/C electrode. At 0.1 A h g^−1^, the initial discharge and charge capacities are 1028.4 and 409.1 mA h g^−1^, with an initial coulomb efficiency (ICE) of 39.8%. However, the TiO_2_ electrode exhibits significantly lower capacities of 749.9 mA h g^−1^ and 223.2 mA h g^−1^, with an ICE of only 29.7% (in Figure S2b). The emergence of irreversible capacities is related to the establishment of the SEI layer and electrolyte decomposition. The higher ICE in the SN-TiO_2_/C electrode indicates fewer side effects due to S/N co-doping.

Figure 3c displays the GCD profiles of the SN-TiO_2_/C electrode, and the cycle stability of the three electrodes at 0.2 A g^−1^ are compared in Figure 3d, which all completed 10 cycles after 0.1 A g^−1^. The TiO_2_, SN-TiO_2_ and SN-TiO_2_/C electrodes exhibit the capacities of 138.6, 205.1 and 294.1 mA h g^−1^ at the 400th cycle, leading to a capacity retention of 88.9%, 89.4% and 90.7%, respectively. All three electrodes demonstrate stable cycling performance. The TiO_2_ electrode exhibits a comparatively lower reversible capacity in contrast to the SN-TiO_2_/C electrode, which achieves the highest reversible capacity. To further validate the impact of S/N co-doping and carbon coating on Na storage performance, the rate capabilities of the three electrodes were assessed. As depicted in Figure 3e,f and Figure S3a,b, the SN-TiO_2_/C electrode demonstrates the reversible capacities of 336.3, 295.6, 264.9, 242.8, 221.3, 192.2 and 164.6 mA h g^−1^ at 0.1 to 0.2, 0.5, 1, 2, 5 and 10 A g^−1^, respectively. Remarkably, it maintains 148.3 mA h g^−1^ at 15 A g^−1^ (~45 C) and even reaches 44% of the initial 0.1 A g^−1^. Upon reverting to 0.1 A g^−1^, the capacity is recovered to 330.4 mAh g^−1^, nearly identical to the initial value, demonstrating the impressive reversibility of the SN-TiO_2_/C electrode. However, the SN-TiO_2_ electrode, without carbon coating, exhibits a moderate rate performance, achieving 211.2 mA h g^−1^ at 0.1 A g^−1^ and decreases to 80.6 mA h g^−1^ at 15 A g^−1^. The TiO_2_ electrode displays a clearly low average capacity of 156.7 mA h g^−1^ at 0.1 A g^−1^, and when increased to 10 A g^−1^, the capacity drops to 58.2 mA h g^−1^. These results indicate that the reversible capacity and rate property of the SN-TiO_2_ electrode were enhanced compared to the TiO_2_ electrode. This enhancement is due to sulfur and nitrogen co-doping, which introduces oxygen vacancies within the TiO_2_ lattice, thereby providing additional Na-ion adsorption sites and facilitating faster electron transport, which is pivotal for increasing the reversible capacity and improved electrochemical kinetics. The SN-TiO_2_/C electrode demonstrates the highest reversible capacity and superior reaction dynamics, attributable to the synergistic effect between sulfur and nitrogen co-doping combined with conductive carbon coating. This indicates that beyond the doping effects, the carbon composite further boosts performance by establishing a highly conductive network that promotes efficient electron transport and additional Na-ion storage capacity. The robust reversible capacity and vigorous reaction dynamics of the SN-TiO_2_/C electrode confirm the efficacy of combined sulfur and nitrogen co-doping and carbon composite strategies in boosting the electrochemical performance. As depicted in Figure 3g, the extended cyclability of SN-TiO_2_/C was tested at 2 A g^−1^ and demonstrates a high capacity of 189.6 mA h g^−1^ after 3000 cycles, maintaining 94% of its initial capacity, indicating an exceptional cycle stability. To our knowledge, the obtained SN-TiO_2_/C electrode delivers significant superiority when compared with previously reported TiO_2_-based materials (Table S1, Supporting Information).

**Figure 4:**
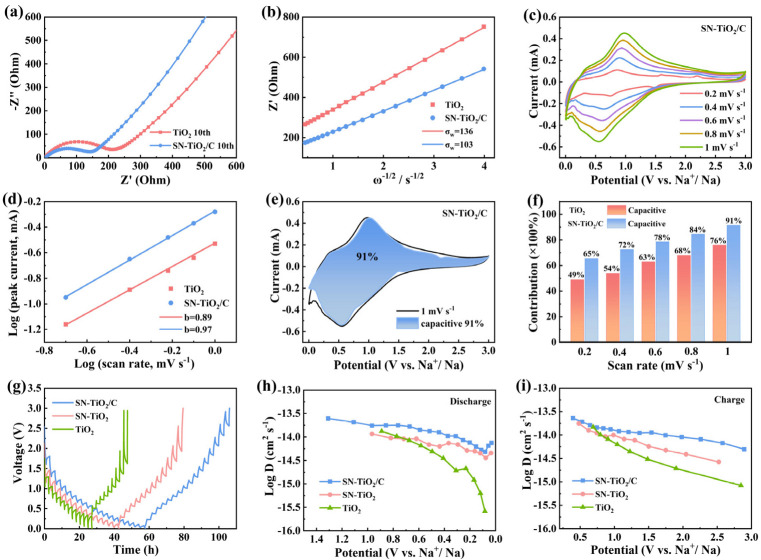
(**a**) EIS profiles of TiO_2_, SN-TiO_2_ and SN-TiO_2_/C and (**b**) correlation between Z′ and ω^−1/2^. (**c**) CV curves of SN-TiO_2_/C electrode. (**d**) Linear correlation of peak currents and scan rates. (**e**) Capacitive contribution of SN-TiO_2_/C electrode at 1.0 mV s^−1^. (**f**) Comparison of the capacitive contributions of the two electrodes. (**g**) GITT profiles and (**h**,**i**) diffusion constants of TiO_2_ and SN-TiO_2_/C electrodes.

To elucidate the diffusion dynamics of Na-ions within the TiO_2_, SN-TiO_2_ and SN-TiO_2_/C electrodes, electrochemical impedance spectroscopy (EIS), CV and Galvanostatic Intermittent Titration Technique (GITT) tests were performed. As illustrated in Figure 4a, the EIS curves comprise a quasi-semicircle shape and a quasi-linear segment. The quasi-semicircle is indicative of the charge-transfer resistance (R_ct_), whereas the quasi-linear segment is indicative of the Na^+^ diffusion process within the electrodes [41]. Clearly, SN-TiO_2_/C presents a markedly lower R_ct_ value compared to the TiO_2_ electrode, indicating that sulfur and nitrogen co-doping, coupled with carbon coating, substantially enhances electronic conductivity and facilitates more efficient charge transfer at the interface. Figure 4b depicts the Z′ − ω^−1/2^ curves, and ω represents the angular frequency (ω = 2πf). The Warburg impedance coefficient (σ_w_) can be calculated based on Equation (1), and a lower σ_w_ value signifies faster Na-ion diffusion kinetics within the electrode [42,43].

Z′ = R_S_ + R_ct_ + σ_w_ω^−1/2^ (1)

Accordingly, the SN-TiO_2_/C anode has a clearly lower σ_w_ value (103 Ω s^−1/2^) than that of TiO_2_ (136 Ω s^−1/2^), corresponding to superior Na^+^ diffuse kinetics in the SN-TiO_2_/C electrode.

Figure 4c, Figure S4a and S5a unravel the CV curves of the three electrodes. The b-values, calculated from the linear relation between the peak current and scan rate, serve as a crucial indicator to distinguish the contribution of pseudocapacitance and diffusion-controlled processes during charge storage. The cathodic b-values (Figure 4d) for the TiO_2_ and SN-TiO_2_/C electrodes are 0.89 and 0.97, respectively. The higher b-value of SN-TiO_2_/C is indicative of fast charge storage kinetics [44]. The pseudocapacitive contribution ratios for the TiO_2_, SN-TiO_2_ and SN-TiO_2_/C electrodes from 0.2 to 1 mV s^−1^ were calculated using the following formula: i = k_1_*v* + k_2_*v*^1/2^ [45], in which k_1_*v* and k_2_*v*^1/2^ are indicative of the surface-dominant and diffusion-controlled mechanisms, respectively. As reflected in Figure 4e,f, Figure S4b and S5b, the capacitive contribution increases progressively with higher scan rates, reaching 76%, 85% and 91% of the TiO_2_, SN-TiO_2_ and SN-TiO_2_/C electrodes at 1 mV s^−1^, respectively. The high capacitive contribution of the SN-TiO_2_/C electrode unravels a surface-predominant pseudocapacitance process for Na storage, which enables it to exhibit exceptional performance in both its high-rate capability and cycling stability. The GITT technique was employed to assess the Na-ion diffusion coefficients and kinetic properties of the electrodes. The diffusion coefficient (D_Na_^+^) can be evaluated in accordance with Fick’s second law [46,47]:

D = 4*l* (ΔE_s_/ΔE_τ_)^2^/πτ (2)

where ΔE_S_ represents the steady-state voltage change, ΔE_*τ*_ denotes the voltage change produced by the continuous current pulse, and τ represents the time of the current pulse. As illustrated in Figure 4h,i, the SN-TiO_2_ electrode shows a moderate rate of D_Na_^+^ values, which is higher than that of the TiO_2_ electrode, suggesting more efficient Na-ion diffusion, ascribed to the defects introduced by S and N co-doping. The SN-TiO_2_/C electrode demonstrates larger D_Na_^+^ values compared to the SN-TiO_2_ electrode, implying the further enhanced Na-ion diffusion kinetics resulting from carbon coating. In summary, these results reveal that sulfur and nitrogen co-doping, along with carbon coating, markedly boosts ionic and electronic conductivity and enhances the Na-ion electrochemical kinetics of the SN-TiO_2_/C electrode. These modifications result in higher specific capacities and robust rate capabilities.

To assess the practicality of SN-TiO_2_/C, a sodium-ion capacitor was constructed. Initially, the electrical double-layer energy storage performances of the AC cathode were assessed. The CV profiles of the AC cathode, depicted in Figure S6a, which display an approximately rectangular shape, are indicative of double-layer capacitance behavior. Figure S4 illustrate the rate capability and the corresponding GCD profiles of AC, demonstrating high rates of capacity of 72.1, 66.4, 60.7, 56.5, 50.8, 43.8 and 34.2 mAh g^−1^ from 0.1 to 10 A g^−1^, respectively. Furthermore, the AC cathode demonstrated superior cyclability, as shown in Figure S4, preserving 95% of its initial capacity after 150 cycles at 0.1 A g^−1^.

**Figure 5:**
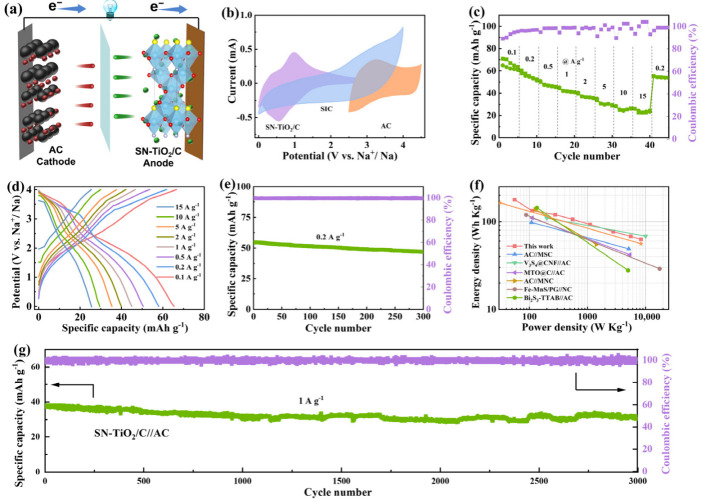
(**a**) Schematic representation and electrochemical properties of the SN-TiO_2_/C//AC SIC. (**b**) CV curves. (**c**,**d**) Rate performance and corresponding GCD curves. (**e**) Cycling stability. (**f**) Ragone plot of the SN-TiO_2_/C//AC device compared to counterparts. (**g**) Cycling capability of the SIC at 2 A g^−1^.

A sodium-ion capacitor (SIC) was constructed, incorporating SN-TiO_2_/C as the anode and utilizing commercial activated carbon (AC) as the cathode. The electrochemical performances of the SN-TiO_2_/C//AC SIC were investigated over 0–4 V. A mass ratio of 1:3 was established between SN-TiO_2_/C and activated carbon (AC) to ensure capacity matching. The discharge process is depicted in Figure 5a. ClO^4−^ ions desorb from the porous surface of the AC cathode, while Na^+^ ions desorb and de-intercalate from the SN-TiO_2_/C anode. As depicted in Figure 5b, the SN-TiO_2_/C//AC device presents asymmetrical CV curves, which indicate the intercalation/deintercalation of Na^+^ ions within the SN-TiO_2_/C anode and the double-layer energy storage mechanism of the AC cathode. Figure 5c,d display the rate performance and GCD profiles of the SN-TiO_2_/C//AC capacitor, which delivers high discharge capacities of 65, 57.9, 50.9, 45, 40.5, 35, 29.5 and 26 mAh g^−1^ from 0.1 to 15 A g^−1^, respectively. As illustrated in Figure 5e, SN-TiO_2_/C//AC manifests a capacity of 47.6 mAh g^−1^ after 300 cycles at 0.2 A g^−1^, retaining 87% of its initial capacity. At a power output of 55.6 W kg^−1^, the SN-TiO_2_/C//AC SIC reaches an impressive energy density of 177.9 W h kg^−1^, maintaining 52.5 Wh kg^−1^, even at 8333 W kg^−1^. The device features extremely short charge and discharge times of 26 and 27 s, respectively, indicating its exceptional rate capability. This superiority is clearly illustrated by the Ragone plot in Figure 5f, which implies the energy and power output of the SN-TiO_2_/C//AC device outperforms many other previously reported sodium-ion capacitors, such as AC//MSC [48], V_3_S_4_@CNF//AC [49], MTO@C//AC [50], AC//MNC [51], Fe-MnS/PG//NC [11], Bi_2_S_3_-TTAB//AC [5] and so on. Additionally, we investigated the long-cycle performance. As depicted in Figure 5g, the SN-TiO_2_/C//AC capacitor exhibits exceptional long-term cycle durability at 2 A g^−1^, maintaining 81% of its capacity after 3000 cycles.

## 3. Materials and Methods

### 3.1. Synthesis of TiO_2_ Nanoplates

Initially, 4 mL of 40% HF solution was added to 20 mL of titanium butoxide (TBOT) with agitation for 1 h. The mixture was then placed in a Teflon-lined autoclave and heated at 180 °C for 18 h. After naturally cooling to ambient temperature, the sample was separated by centrifugation. The sample was then immersed in 0.1 M of NaOH aqueous solution for 12 h and subsequently rinsed three times with deionized water. Finally, the TiO_2_ nanoplate precursor sample was dried at 80 °C for 12 h.

### 3.2. Synthesis of TiO_2_/PDA Nanoplates

First, 0.2 g of the TiO_2_ nanoplate precursor was dispersed in 100 mL of 0.01 M Tris (trimethylol aminomethane) solution. After stirring at ambient temperature for 2 h, 40 mg of dopamine hydrochloride (DA) was introduced into the solution. The solution was then stirred for 24 h at ambient temperature. Finally, the TiO_2_/PDA nanoplates were separated by centrifugation, rinsed three times with deionized water and dried at 80 °C for 12 h.

### 3.3. Synthesis of SN-TiO_2_/C Nanoplates

In the synthesis of SN-TiO_2_/C nanoplates, 0.2 g of TiO_2_/PDA nanoplate precursor and thiourea were placed on the downstream side and upstream side in the chamber of the furnace, respectively. Subsequently, the chamber was then heated to 550 °C for 2 h. During this process, the decomposition of thiourea released ammonia and hydrogen sulfide, which acted as nitrogen and sulfur sources for doping titanium dioxide. The PDA was transformed into sulfur- and nitrogen-doped carbon, leading to the formation of a nitrogen and sulfur co-doped and carbon-coated TiO_2_ sample (SN-TiO_2_/C). For comparison, the TiO_2_ nanoplate precursor was annealed at 550 °C for 2 h under an Ar atmosphere to obtain a TiO_2_ nanoplate sample, denoted as TiO_2_. The TiO_2_ nanoplate precursor and thiourea were annealed under the same conditions to obtain a nitrogen- and sulfur-doped TiO_2_ sample (SN-TiO_2_).

## 4. Conclusions

In summary, we constructed a unique sodium-ion capacitor that delivers an impressive energy density of 177.9 W h kg^−1^, utilizing sulfur and nitrogen co-doped titanium dioxide/carbon (SN-TiO_2_/C) as the anode and activated carbon (AC) as the cathode. The SN-TiO_2_/C anode, characterized by an ultrathin structure, is engineered through an integration of structural refinement and heteroatom doping, with carbon coating. The ultrathin nanoplates structure maximizes the available sodium-ion adsorption sites, while sulfur- and nitrogen-doping-induced defects enhance electrical conductivity and provide additional Na-ion adsorption sites. These synergistic modifications markedly enhance the reversible capacity (336.3 mA h g^−1^ at 0.1 A g^−1^) and improve the electrochemical kinetics of Na-ion storage in SN-TiO_2_/C, thereby facilitating their application in sodium-ion capacitors.

## Data Availability

The data presented in this study are available on request from the corresponding author. The data are not publicly available due to institutional restriction.

## Supplementary Materials

The following supporting information can be downloaded at: https://www.mdpi.com/article/10.3390/molecules29184507/s1, Figure S1: SEM images of SN-TiO_2_/C. Figure S2: (a) CV curves of TiO_2_ electrode at 0.2 mV s^−1^. (b) Initial GCD profiles of TiO_2_ at 0.1 A g^−1^. Figure S3: (a) The GCD profiles of TiO_2_ and SN-TiO_2_ electrodes. Figure S4: (a) CV curves at various sweep rates of TiO_2_ electrode. (b) Capacitive contribution of TiO_2_ electrode at a sweep rate of 1.0 mV s^−1^. Figure S5: (a) CV curves at various sweep rates of SN-TiO_2_ electrode. (b) Capacitive contribution of SN-TiO_2_ electrode at a sweep rate of 1.0 mV s^−1^. Figure S6: The electrochemical properties of the AC cathode in half cell: (a) CV curves of the AC cathode at scan rate from 0.5 to 10 mV s^−1^, within a voltage range of 2.5–4.5 V (vs Na/Na^+^). (b) Rate capabilities and (c) the corresponding GCD profiles of AC cathode. (d) Cycle performances of AC cathode at 0.1 A g^−1^. Table S1: Electrochemical performances of TiO_2_-based materials for SIBs anodes comparison with the previously reported in the literature.

## References

[1] Li H., He Y., Wang Q., Gu S., Wang L., Yu J., Zhou G., Xu L. (2023). SnSe_2_/NiSe_2_@N-doped carbon yolk-shell heterostructure construction and selenium vacancies engineering for ultrastable sodium-ion storage. Adv. Energy Mater..

[2] Li J., He Y., Dai Y., Zhang H., Zhang Y., Gu S., Wang X., Gao T., Zhou G., Xu L. (2024). Heterostructure interface construction of cobalt/molybdenum selenides toward ultra-stable sodium-ion half/full batteries. Adv. Funct. Mater..

[3] Yuan J., Qiu M., Hu X., Liu Y., Zhong G., Zhan H., Wen Z. (2022). Pseudocapacitive vanadium nitride quantum dots modified one-dimensional carbon cages enable highly kinetics-compatible sodium ion capacitors. ACS Nano.

[4] Cai J., Wang L., Tao S., Liu Y., Cao Z., Song Z., Xiao X., Zhu Y., Deng W., Hou H. (2023). Electrochemistry enabled heterostructure with high tap density for ultrahigh power Na-ion capacitors. Adv. Energy Mater..

[5] Xiao Y., Jiang H., Zhang K., Kong Y., Zhang S., Wang H., Yuan G., Su D., Zhou J., Wang X. (2024). Strain engineering of Bi_2_S_3_ microspheres via organic intercalation enabled high performance sodium storage. Chem. Eng. J..

[6] He Y., Liu D., Jiao J., Liu Y., He S., Zhang Y., Cheng Q., Fang Y., Mo X., Pan H. (2024). Pyridinic N-dominated hard carbon with accessible carbonyl groups enabling 98% initial coulombic efficiency for sodium-ion batteries. Adv. Funct. Mater..

[7] Yang Z., Liu X., Ma X., Cao T., Xu J., Feng H., Diao R., Qi F., Huang H., Ma P. (2024). Efficient preparation of biomass-based ultra-thin 2D porous carbon materials by in situ template-activation and its application in sodium ion capacitors. Adv. Funct. Mater..

[8] Li C., Song Z., Liu M., Lepre E., Antonietti M., Zhu J., Liu J., Fu Y., López-Salas N. (2024). Template-induced graphitic nanodomains in nitrogen-doped carbons enable high-performance sodium-ion capacitors. Energy Storage Mater..

[9] Jiang Y., Han R., Dong J., Yu R., Tan S., Xiong F., Wei Q., Wang J., Cui L., Tian H. (2023). Uncovering the origin of surface-redox pseudocapacitance of molybdenum phosphides enables high-performance flexible sodium-ion capacitors. Chem. Eng. J..

[10] Cai J., Zhou Y., Tao S., Liu Y., Deng W., Hou H., Zou G., Ji X. (2024). Nanocrystalline heterostructure with low voltage hysteresis for ultrahigh-power sodium-ion capacitors. Energy Storage Mater..

[11] Chen Y., Li S., Chen J., Gao L., Guo P., Wei C., Fu J., Xu Q. (2024). Sulfur-bridged bonds enabled structure modulation and space confinement of MnS for superior sodium-ion capacitors. J. Colloid Interf. Sci..

[12] Li T., Zhao D., Du B., Yin Q., Li Y., Xue X., Wei F., Qi J., Sui Y. (2023). Defect-induced electron rich nanodomains in CoSe_0.5_S_1.5_/GA realize fast ion migration kinetics as sodium-ion capacitor anode. J. Energy Chem..

[13] Li Z., Wu R., Zhu Z., Zhu Y., Wang Y., Xu S., Kong Q., Chen J.S. (2024). Improving the electronegativity of N-doped carbon by encapsulating CoFe alloy clusters with a chainmail-like structure for high-energy sodium-ion capacitors. J. Mater. Chem. A.

[14] Han M., Zou Z., Liu J., Deng C., Chu Y., Mu Y., Zheng K., Yu F., Wei L., Zeng L. (2024). Pressure-induced defects and reduced size endow TiO_2_ with high capacity over 20 000 cycles and excellent fast-charging performance in sodium ion batteries. Small.

[15] Wei Q., Chang X., Butts D., DeBlock R., Lan K., Li J., Chao D., Peng D.L., Dunn B. (2023). Surface-redox sodium-ion storage in anatase titanium oxide. Nat. Commun..

[16] Li T., Kong L.Y., Bai X., Wang Y.X., Qi Y.X. (2023). Promoting amorphization of commercial TiO_2_ upon sodiation to boost the sodium storage performance. J. Energy Chem..

[17] Kang M., Ruan Y., Lu Y., Luo L., Huang J., Zhang J.M., Hong Z. (2019). An interlayer defect promoting the doping of the phosphate group into TiO_2_(B) nanowires with unusual structure properties towards ultra-fast and ultra-stable sodium storage. J. Mater. Chem. A.

[18] Fu L., Wang Q., He H., Tang Y., Wang H., Xie H. (2021). Dual carbon coating engineering endows hollow structured TiO_2_ with superior sodium storage performance. J. Power Sources.

[19] Diao Z., Wang Y., Zhao D., Zhang X., Mao S.S., Shen S. (2021). Ultra-small TiO_2_ nanoparticles embedded in carbon nanosheets for high-performance sodium storage. Chem. Eng. J..

[20] Li B., Anwer S., Huang X., Luo S., Fu J., Liao K. (2021). Nitrogen-doped carbon encapsulated in mesoporous TiO_2_ nanotubes for fast capacitive sodium storage. J. Energy Chem..

[21] Lin D., Wang M., Weng Q., Qin X., An L., Chen G., Liu Q. (2023). Three dimensional titanium dioxide nanotube arrays induced nanoporous structures and stable solid electrolyte interphase layer for excellent sodium storage in ether-based electrolyte. J. Power Sources.

[22] Chen J., Fu Y., Sun F., Hu Z., Wang X., Zhang T., Zhang F., Wu X., Chen H., Cheng G. (2020). Oxygen vacancies and phase tuning of self-supported black TiO_2-X_ nanotube arrays for enhanced sodium storage. Chem. Eng. J..

[23] Yang J., Huang M., Xu L., Xia X., Peng C. (2022). Self-assembled titanium-deficient undoped anatase TiO_2_ nanoflowers for ultralong-life and high-rate Li^+^/Na^+^ storage. Chem. Eng. J..

[24] Luo B., Wang W., Wang Q., Ji W., Yu G., Liu Z., Zhao Z., Wang X., Wang S., Zhang J. (2023). Facilitating ionic conductivity and interfacial stability via oxygen vacancies-enriched TiO_2_ microrods for composite polymer electrolytes. Chem. Eng. J..

[25] Wang C., Zhang J., Wang X., Lin C., Zhao X.S. (2020). Hollow rutile cuboid arrays grown on carbon fiber cloth as a flexible electrode for sodium-ion batteries. Adv. Funct. Mater..

[26] Wang C., Yao Q., Wang M., Zheng C., Wang N., Bai Z., Yang J., Dou S., Liu H. (2023). Highly conductive hierarchical TiO_2_ micro-sheet enables thick electrodes in sodium storage. Adv. Funct. Mater..

[27] Guan S., Fan Q., Shen Z., Zhao Y., Sun Y., Shi Z. (2021). Heterojunction TiO_2_@TiOF_2_ nanosheets as superior anode materials for sodium-ion batteries. J. Mater. Chem. A.

[28] He T., An Q., Zhang M., Kang N., Kong D., Song H., Wu S., Wang Y., Hu J., Zhang D. (2024). Multiscale interface engineering of sulfur-doped TiO_2_ anode for ultrafast and robust sodium storage. ACS Nano.

[29] Chen J., Zhu K., Liang P., Rao Y., Li X., Zheng H., Yan K., Wang J., Liu J. (2023). Metal-organic framework derived S-doped anatase TiO_2_@C to store Na^+^ with high-rate and long-cycle life. J. Alloy. Compd..

[30] Cai Q., Li X., Hu E., Wang Z., Lv P., Zheng J., Yu K., Wei W., Ostrikov K. (2022). Overcoming ion transport barrier by plasma heterointerface engineering: Epitaxial titanium carbonitride on nitrogen-doped TiO_2_ for high-performance sodium-ion batteries. Small.

[31] Chen D., Wu Y., Huang Z., Chen J. (2022). A novel hybrid point defect of oxygen vacancy and phosphorus doping in TiO_2_ anode for high-performance sodium ion capacitor. Nano-Micro Lett..

[32] Yao T., Wang H., Ji X., Wang D., Zhang Q., Meng L., Shi J.W., Han X., Cheng Y. (2023). Introducing hybrid defects of silicon doping and oxygen vacancies into MOF-derived TiO_2–X_@carbon nanotablets toward high-performance sodium-ion storage. Small.

[33] Feng W., Meng C., Guo X., Wu B., Sui X., Wang Z. (2024). Defect-driven reconstruction of Na-ion diffusion channels enabling high-performance Co-doped TiO_2_ anodes for Na-ion hybrid capacitors. Adv. Energy Mater..

[34] Fan M., Lin Z., Zhang P., Ma X., Wu K., Liu M., Xiong X. (2020). Synergistic effect of nitrogen and sulfur dual-doping endows TiO_2_ with exceptional sodium storage performance. Adv. Energy Mater..

[35] Wang Q., He H., Luan J., Tang Y., Huang D., Peng Z., Wang H. (2019). Synergistic effect of N-doping and rich oxygen vacancies induced by nitrogen plasma endows TiO_2_ superior sodium storage performance. Electrochim. Acta.

[36] Qu Y., Zhu S., Dong X., Huang H., Qi M. (2021). Nitrogen-doped TiO_2_ nanotube anode enabling improvement of electronic conductivity for fast and long-term sodium storage. J. Alloy. Compd..

[37] Zhou X., Huang X., He S., Lu Y., Shen X., Tang S. (2024). In situ construction of (NiCo)_3_Se_4_ nanobeads embedded in n-doped carbon 3D interconnected networks for enhanced sodium storage. Inorg. Chem..

[38] He H., He J., Yu H., Zeng L., Luo D., Zhang C. (2023). Dual-interfering chemistry for soft-hard carbon translation toward fast and durable sodium storage. Adv. Energy Mater..

[39] Du Y., Fan H., Bai L., Song J., Jin Y., Liu S., Li M., Xie X., Liu W. (2023). Molten salt-assisted construction of hollow carbon spheres with outer-order and inner-disorder heterostructure for ultra-stable potassium ion storage. ACS Appl. Mater. Interfaces.

[40] Zhao H., Zhong J., Qi Y., Liang K., Li J., Huang X., Chen W., Ren Y. (2023). 90 C fast-charge Na-ion batteries for pseudocapacitive faceted TiO_2_ anodes based on robust interface chemistry. Chem. Eng. J..

[41] Li J., Hu G., Yu R., Liao X., Zhao K., Li T., Zhu J., Chen Q., Su D., Ren Y. (2023). Revolutionizing lithium storage capabilities in TiO_2_ by expanding the redox range. ACS Nano.

[42] Zhu K., Gao S., Bai T., Li H., Zhang X., Mu Y., Guo W., Cui Z., Wang N., Zhao Y. (2024). Heterogeneous MoS_2_ nanosheets on porous TiO_2_ nanofibers toward fast and reversible sodium-ion storage. Small.

[43] Zhang X., Wang J., Jiang Y., Zhang M., Min H., Yang H., Wang J. (2024). A 3D crinkled MXene/TiO_2_ heterostructure with interfacial coupling for ultra-fast and reversible potassium storage. J. Mater. Chem. A.

[44] Chen Z., Yu Z., Wang L., Huang Y., Huang H., Xia Y., Zeng S., Xu R., Yang Y., He S. (2023). Oxygen defect engineering toward zero-strain V_2_O_2.8_@porous reticular carbon for ultrastable potassium storage. ACS Nano.

[45] Pan Z., Qian Y., Li Y., Xie X., Lin N., Qian Y. (2023). Novel bilayer-shelled N, O-doped hollow porous carbon microspheres as high performance anode for potassium-ion hybrid capacitors. Nano-Micro Lett..

[46] Yuan X., Xiong Y., Liu Y., Wei X., Wei F., Wang M., Cao Y., Tao G., Zhang Q., Wan Q. (2023). Regulation of the surface activity of carbon anodes for rationalization of potassium storage. Chem. Commun..

[47] Pei Y.R., Zhou H.Y., Zhao M., Li J.C., Ge X., Zhang W., Yang C.C., Jiang Q. (2023). High-efficiency sodium storage of Co_0.85_Se/WSe_2_ encapsulated in N-doped carbon polyhedron via vacancy and heterojunction engineering. Carbon Energy.

[48] Zhang H., Liu B., Lu Z., Hu J., Xie J., Hao A., Cao Y. (2023). Sulfur-bridged bonds heightened Na-storage properties in MnS nanocubes encapsulated by S-doped carbon matrix synthesized via solvent-free tactics for high-performance hybrid sodium ion capacitors. Small.

[49] Mao Z.F., Shi X.J., Zhang T.Q., Liang P.J., Wang R., Jin J., He B.B., Gong Y.S., Wang Q., Tong X.L. (2023). Mechanically flexible V_3_S_4_@carbon composite fiber as a high-capacity and fast-charging anode for sodium-ion capacitors. Rare Met..

[50] Chen C., Li N.W., Zhang X.Y., Zhang C.H., Qiu J., Yu L. (2022). Interlayer-expanded titanate hierarchical hollow spheres embedded in carbon nanofibers for enhanced Na storage. Small.

[51] Zhang H., Liu B., Wang S., Yuan C., Lu Z., Hu J., Xie J., Cao Y. (2024). 2D heterostructural Mn_2_O_3_ quantum dots embedded N-doped carbon nanosheets with strongly stable interface enabling high-performance sodium-ion hybrid capacitors. J. Colloid Interf. Sci..

