# Supplementary material for: S/N Co-Doped Ultrathin TiO2 Nanoplates as an Anode Material for Advanced Sodium-Ion Hybrid Capacitors
Source: Molecules. 2024 Sep 23;29(18):4507. doi: 10.3390/molecules29184507 (PMC11433928; doi:10.3390/molecules29184507)
Supplement: Supplementary file 1 [file molecules-29-04507-s001.zip › molecules-3182080-supplementary.pdf]

Supporting Information

Experimental section

1. Materials Characterization

The crystal structure and chemical composition of the samples were analyzed using various techniques, including X-ray diffraction (XRD, Bruker AXS D8-Focus, Cu Kα radiation), X-ray photoelectron spectroscopy (XPS, VG Multilab2000), Raman microscopy (Renishaw RM-1000, 532 nm excitation) and atomic force microscopy (AFM, Bruker Dimension Icon). The surface morphology and structure were investigated using field emission scanning electron microscopy (FE-SEM, Hitachi SU8010) and transmission electron microscopy (TEM, Philips CM12 TEM/STEM).

2. Electrochemical characterizations

All electrochemical tests were conducted using CR2032-type coin cells. For the working electrode, the TiO_2, SN-TiO_2 and SN-TiO_2/C samples were mixed with super P, and polyvinylidene difluoride (PVDF) in a weight ratio of 8:1:1 to form a slurry in N-methyl-2-pyrrolidone (NMP). The slurry was cast onto a copper foil and dried in a vacuum oven at 100 °C for 12 hours. The electrolyte used 1 M $NaClO_4$ in propylene carbonate (PC), with a glass fiber membrane (Whatman, GF/D) and sodium metal foil served as the separator and counter electrodes, respectively. All cells were assembled in an argon-filled glovebox. Galvanostatic charge-discharge measurements were carried out within a cutoff voltage range of 0.01-3.0 V vs. Na/Na^+ using a CT2001A cell test instrument (LAND Electronic Co.). Cyclic voltammetry (CV) studies were carried out on an electrochemical workstation (CH Instruments, model 660C). Electrochemical impedance spectroscopy (EIS) tests were conducted after 10 cycles at a current density of 0.1 A g^{-1}. The galvanostatic intermittent titration technique (GITT) was performed with a pulse current of 100 mA g^{-1} for 1 h, followed by rest intervals of 2 h. The power densities (P, W kg^{-1}) and energy densities (E, W h kg^{-1}) were calculated using the following equations:

$$E = \int_{t_1}^{t_2} IV dt \qquad \text{(S1)}$$

$$P = E/t \tag{S2}$$

where I represents the charge/discharge current (A), t is the discharge duration (s), V max (4.0 V) and V min (0 V) are the starting and final discharge voltages (V), m is the combined mass of the active material in both the anode and cathode (g), C is the specific capacitance (F g^{-1}), E denotes the energy density (W h kg^{-1}), and P represents the power density (W kg^{-1}).

Supporting Figures

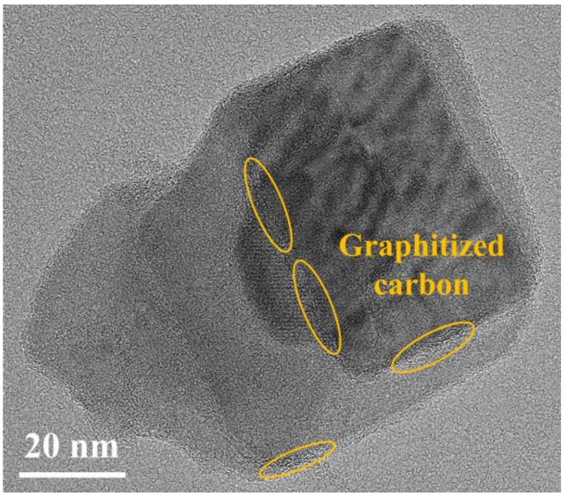

Figure S1. SEM images of SN-TiO_2/C.

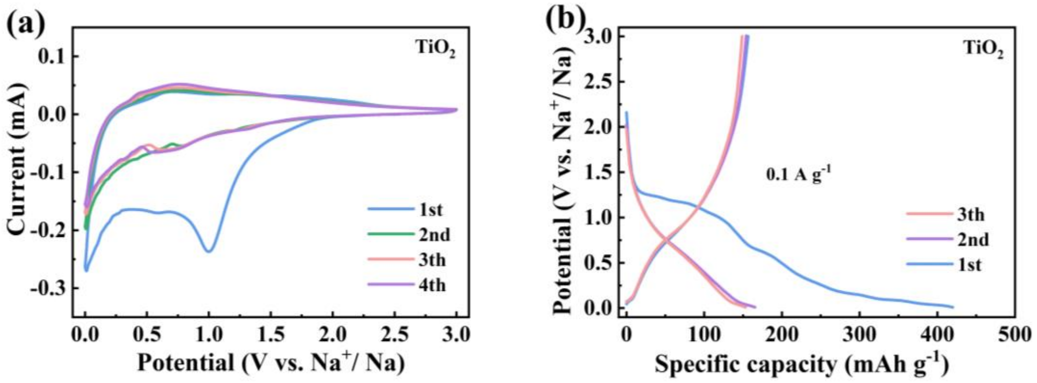

Figure S2. (a) CV curves of TiO_2 electrode at 0.2 mV s^{-1}. (b) Initial GCD profiles of TiO_2 at 0.1 A g^{-1}.

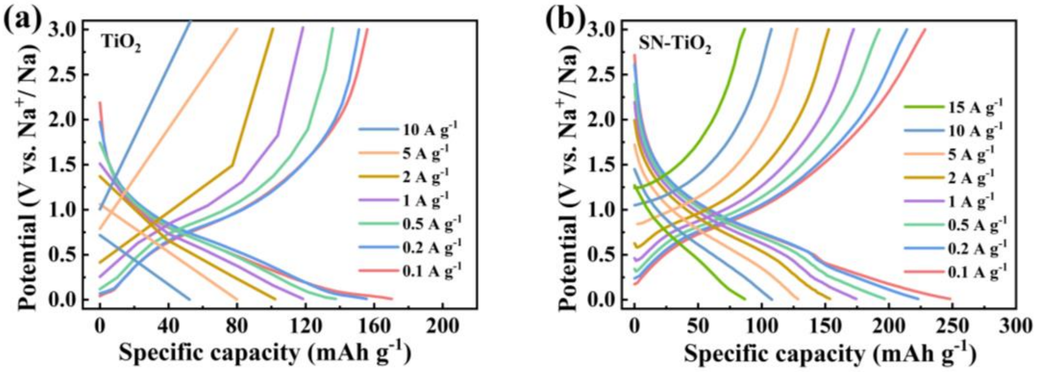

Figure S3. (a) The GCD profiles of TiO_2 and SN-TiO_2 electrodes.

Table S1. Electrochemical performances of TiO_2-based materials for SIBs anodes comparison with the previously reported in the literature.

Samples	Rate capacity/current density (mAh g^{-1}/ A g^{-1})	Capacity/current density/cycles (mAh g^{-1}/ A g^{-1}/ cycles)	Ref.
SN-TiO_2/C	336.3/0.1<br>148.3/15	294.1/0.2/400<br>189.6/2/3000	This work
p-TiO_2@NC	420/0.067<br>170/10	135/0.067/10000	[1]
S-TiO_2@C	283.6/0.1<br>167.7/5	285.1/0.2/200	[2]
TiC_xN_{1-x}/N-TiO_2	358.0/0.0335<br>173.7/3.35	171.1/3.35/600	[3]
N-TiO_{2-x}	265.6/0.05	200/0.1/250	[4]

	147.9/1		
N-TiO_2-NTs	232/0.0335<br>114/3.35	101.1/3.35/500	[5]
TiO_2@$TiOF_2$-36 h	211.2/0.1<br>115.4/5	141.8/0.5/2000	[6]
H–C@TiO_2@C	335.4/0.5<br>124.2/8	256.2/0.2/200	[7]

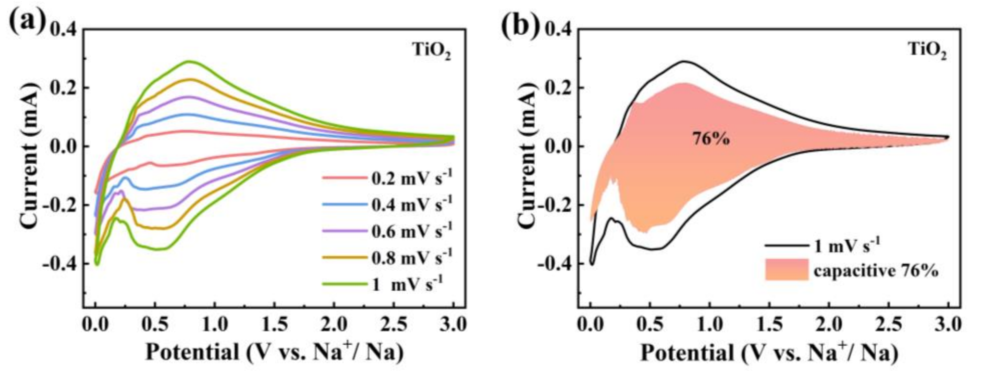

Figure S4. (a) CV curves at various sweep rates of TiO_2 electrode. (b) Capacitive contribution of TiO_2 electrode at a sweep rate of 1.0 mV s^{-1}.

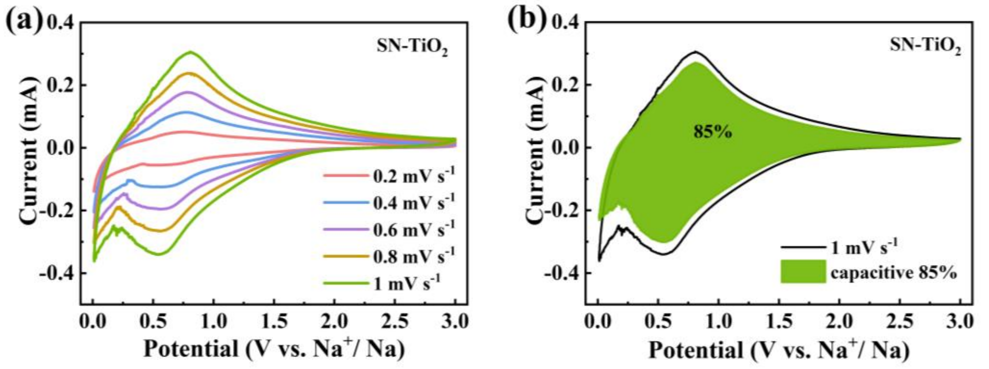

Figure S5. (a) CV curves at various sweep rates of SN-TiO_2 electrode. (b) Capacitive contribution of SN-TiO_2 electrode at a sweep rate of 1.0 mV s^{-1}.

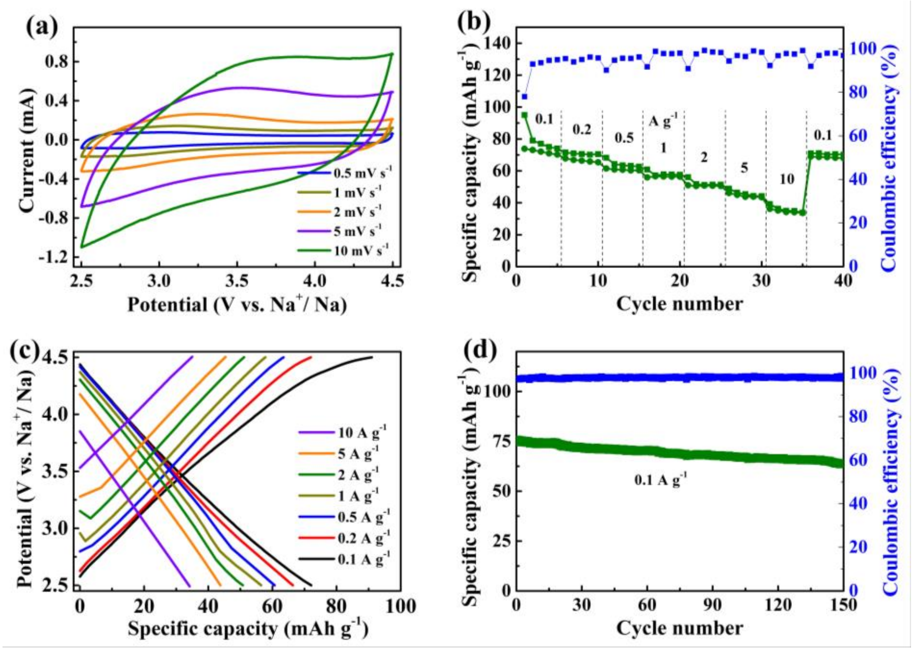

Figure S6. The electrochemical properties of the AC cathode in half cell: (a) CV curves of the AC cathode at scan rate from 0.5 to 10 mV s^{-1}, within a voltage range of 2.5–4.5 V (vs Na/Na^{+}). (b) Rate capabilities and (c) the corresponding GCD profiles of AC cathode. (d) Cycle performances of AC cathode at 0.1 A g^{-1}.

References

1. Zhao, H.; Zhong, J.; Qi, Y.; Liang, K.; Li, J.; Huang, X.; Chen, W., and Ren, Y., 90 C fast-charge Na-ion batteries for pseudocapacitive faceted TiO_2 anodes based on robust interface chemistry, Chem. Eng. J. **2023**, 465, 143032.
2. Chen, J.; Zhu, K.; Liang, P.; Rao, Y.; Li, X.; Zheng, H.; Yan, K.; Wang, J., and Liu, J., Metal-organic framework derived S-doped anatase TiO_2@C to store Na^{+} with high-rate and long-cycle life, Journal of Alloys and Compounds **2023**, 969, 172395.
3. Cai, Q.; Li, X.; Hu, E.; Wang, Z.; Lv, P.; Zheng, J.; Yu, K.; Wei, W., and Ostrikov, K., Overcoming Ion transport barrier by plasma heterointerface engineering: epitaxial titanium carbonitride on nitrogen-doped TiO_2 for high-performance sodium-ion batteries, Small **2022**, 18, 2200694.
4. Wang, Q.; He, H.; Luan, J.; Tang, Y.; Huang, D.; Peng, Z., and Wang, H., Synergistic effect of N-doping and rich oxygen vacancies induced by nitrogen plasma endows TiO_2 superior sodium storage performance, Electrochimica Acta **2019**, 309, 242-252.
5. Qu, Y.; Zhu, S.; Dong, X.; Huang, H., and Qi, M., Nitrogen-doped TiO_2 nanotube anode enabling improvement of electronic conductivity for fast and long-term sodium storage, Journal of Alloys and Compounds **2021**, 889, 161612.
6. Guan, S.; Fan, Q.; Shen, Z.; Zhao, Y.; Sun, Y., and Shi, Z., Heterojunction TiO_2@$TiOF_2$ nanosheets as superior anode materials for sodium-ion batteries, J. Mater. Chem. A **2021**, 9, 5720-5729.
7. Fu, L.; Wang, Q.; He, H.; Tang, Y.; Wang, H., and Xie, H., Dual carbon coating engineering endows hollow structured TiO_2 with superior sodium storage performance, J. Power Sources **2021**, 489, 229516.
